# Case Report: A Chinese woman with primary Sjögren’s syndrome presented with simultaneous involvement of the cerebral and coronary arteries as initial symptoms

**DOI:** 10.3389/fimmu.2025.1696571

**Published:** 2025-11-10

**Authors:** JiaQi Tang, Ting Shen, ZeXian Xu, ChenChen Wang

**Affiliations:** 1 Department of Cardiovascular, Quzhou KeCheng People’s Hospital, Quzhou, Zhejiang, China; 2 Department of Pathology, Quzhou KeCheng People’s Hospital, Quzhou, Zhejiang, China; 3 Department of Radiology, Quzhou KeCheng People’s Hospital, Quzhou, Zhejiang, China; 4 Department of Pharmacy, Quzhou KeCheng People’s Hospital, Quzhou, Zhejiang, China

**Keywords:** primary Sjögren’s syndrome, cerebral artery stenosis, coronary artery stenosis, risk factor, case report

## Abstract

**Introduction:**

Primary Sjögren’s syndrome (pSS) is a heterogeneous autoimmune disorder that may be associated with systemic manifestations such as pulmonary or articular involvement.Compared to other autoimmune diseases, vascular injury and accelerated atherosclerosis in pSS are less studied.

**Case presentation:**

A 51-year-old woman presented with a 2-month history of dizziness, chest tightness, and exertional dyspnea. Vascular imaging revealed multifocal stenoses in the left anterior descending coronary artery (70%), bilateral middle cerebral arteries, right anterior cerebral artery, and left posterior cerebral artery. Her only traditional cardiovascular risk factor was hypertension, and she reported only mild xerostomia. Serology showed anti-SSA positivity. Schirmer’s test was abnormal, and a labial gland biopsy confirmed focal lymphocytic sialadenitis (≥1 foci/4 mm²), leading to a pSS diagnosis per 2016 ACR-EULAR criteria. She responded favorably to a combination of immunomodulators (hydroxychloroquine and total glucosides of paeony), antiplatelet therapy, and statins, with symptom resolution at follow-up.

**Conclusion:**

This case represents an exceptionally rare documented instance of pSS simultaneously affecting both cerebral and coronary arterial systems. It underscores that severe multifocal arterial stenosis can present as the initial manifestation of this disease, even in the absence of prominent dryness symptoms. This highlights the necessity for vascular assessment in pSS patients and the urgency of conducting autoimmune evaluations in cases of unexplained multivessel arteriopathy.

## Introduction

Primary Sjögren’s syndrome (pSS) is a systemic autoimmune rheumatic disease that primarily targets the exocrine glands. However, 30–40% of patients may develop systemic manifestations (1). Multiple previous studies have confirmed the presence of endothelial dysfunction and signs of premature functional impairment of the arterial wall in individuals with pSS (2, 3). In 2018, Yong et al. conducted a meta-analysis in which data were extracted from 10 studies involving a total of 165,291 participants for qualitative synthesis. The pooled results indicated that, compared with the control group, patients with pSS had a significantly increased overall risk of cardiovascular or cerebrovascular events, with an odds ratio (OR) = 1.28 (95% confidence interval 1.11–1.46; P < 0.01) (4). In this report, we describe an exceptionally rare case of pSS in China presenting with vertigo and chest tightness as initial symptoms, accompanied by involvement of both cerebral and coronary arteries. We hope that this case will further enhance rheumatologists’ awareness and vigilance regarding vascular pathology associated with pSS.

## Case description

**Figure 1:**
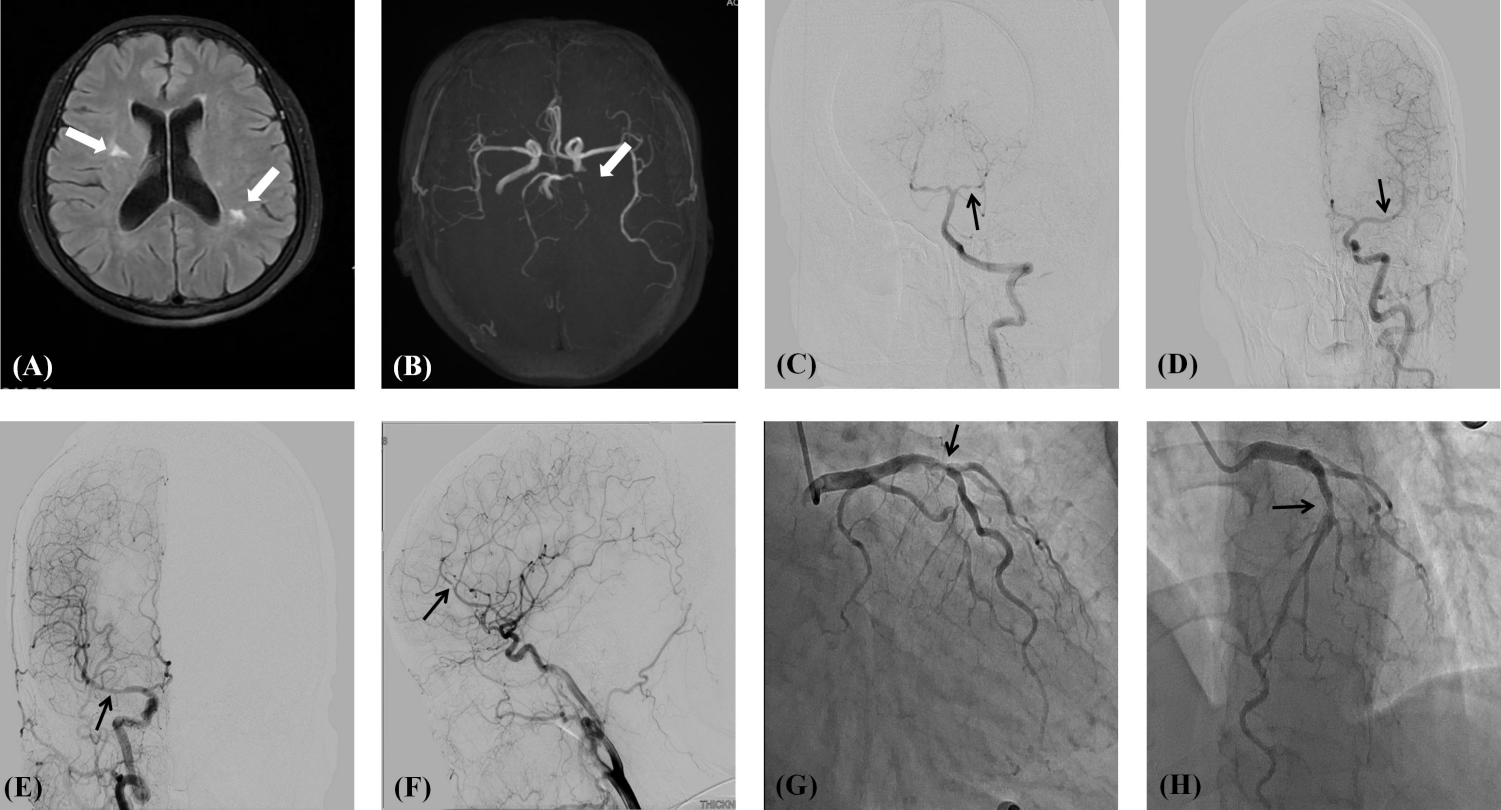
**(A)** Demyelinating changes in the parietal white matter adjacent to the lateral ventricles on both sides; **(B)** Distal stenosis of the left posterior cerebral artery is suspected. **(C)** Severe stenosis of the P1 segment of the left posterior cerebral artery; **(D)** Mild stenosis of the M1 segment of the left middle cerebral artery; **(E)** Moderate stenosis of the middle cerebral artery M1 segment on the right side; **(F)** Severe stenosis of segment A2 of the right anterior cerebral artery; **(G, H)** Proximal segment of the left anterior descending artery shows 70% stenosis.

A 51-year-old female patient was referred to our hospital due to dizziness accompanied by chest tightness and shortness of breath after physical activity for two months. Carotid CTA performed at an outside hospital revealed a hypodense linear septum within the lumen of the right internal carotid artery at its clinoid segment. The findings were suggestive of an intimal ulcer or an early dissection. Brain MRI and MRA revealed demyelinating changes in the bilateral frontal lobes and periventricular regions. Possible distal stenosis of the left posterior cerebral artery was noted (**Figures 1A, B**).

**Figure 2:**
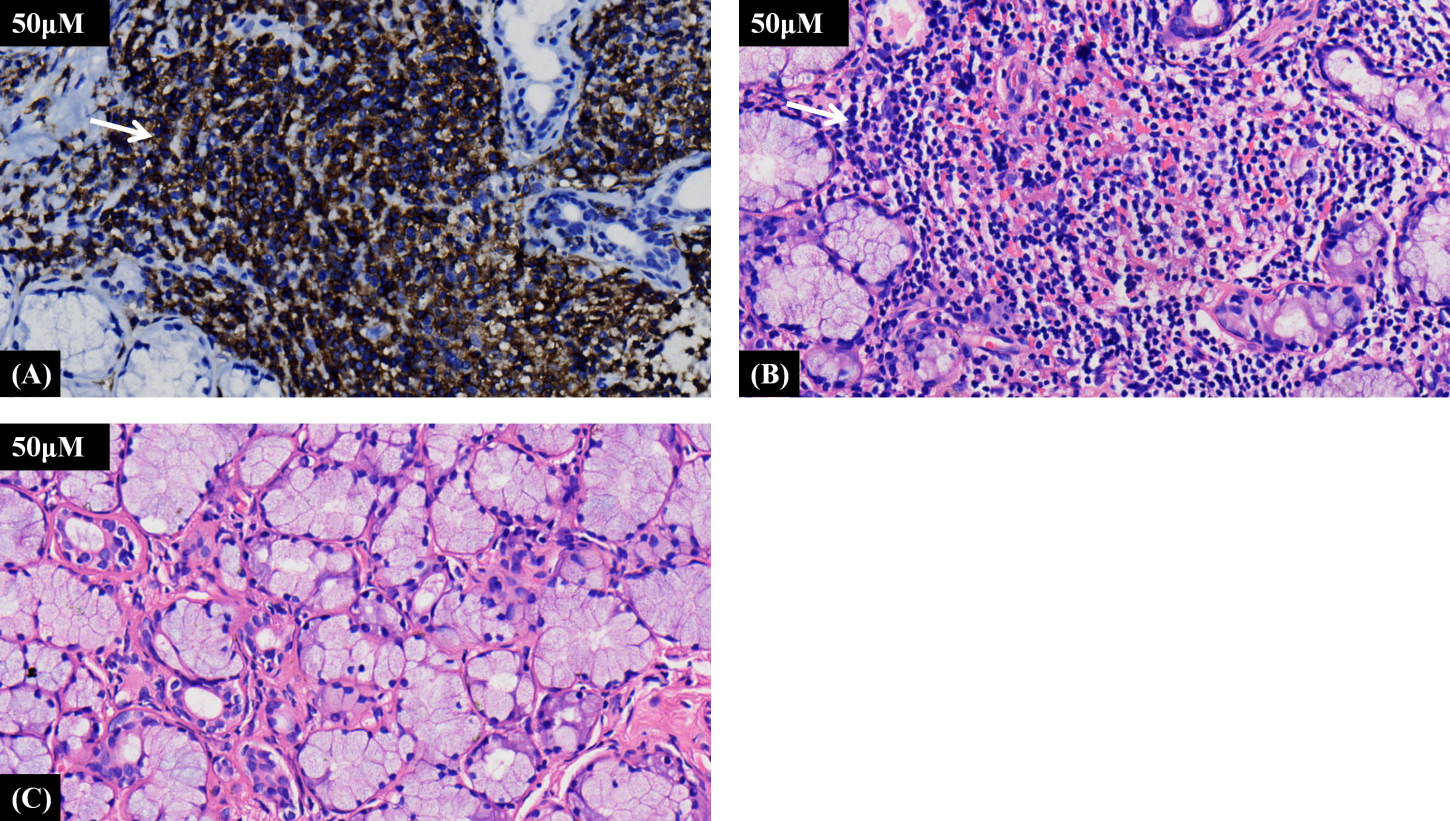
**(A)** Representative photomicrograph of immunohistochemical staining demonstrating lymphocyte infiltration. **(B)** Representative photomicrograph of hematoxylin and eosin staining showing the distribution of lymphocyte infiltration. **(C)** Representative photomicrograph of normal labial salivary gland tissue from the control group. A focus is ≥50 lymphocytes, and the focus score is the number of such foci per 4 mm² (≥1 is diagnostic).

**Figure 3:**
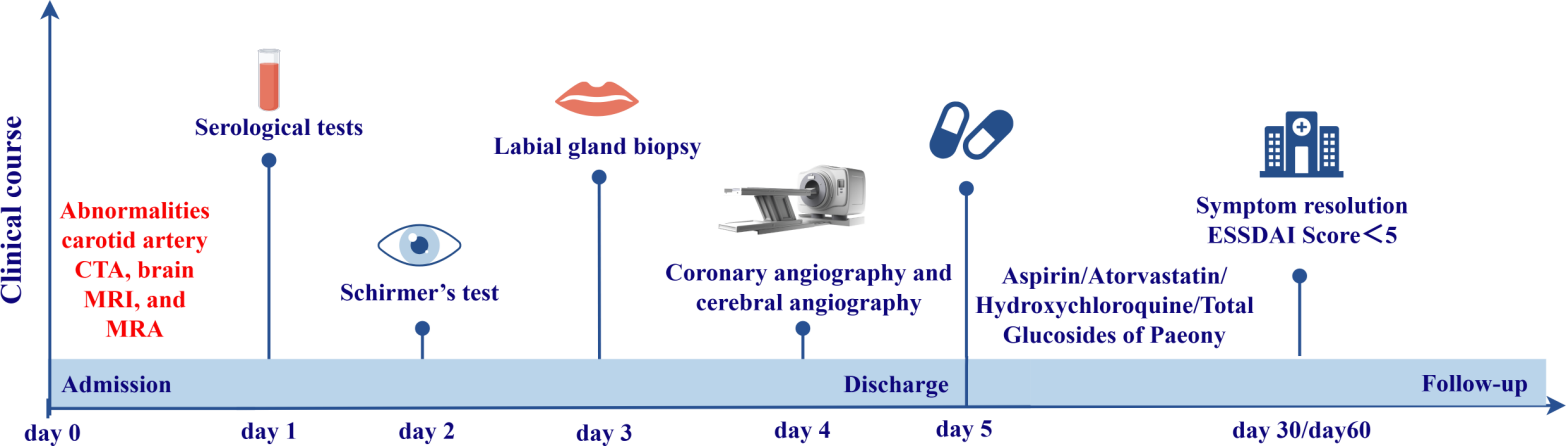
The detailed timeline after the patient’s admission.

Further history taking during hospitalization revealed that the patient had a history of mild dry mouth and polydipsia, without dry eyes. The patient could ingest dry solid foods without the need for water assistance and had no dental caries. The antinuclear antibody titer was 1:80 (Chemiluminescent Immunoassay), along with other serological indicators as shown in **Table 1**. Schirmer’s test results were 10 mm/5 min in the left eye and 4 mm/5 min in the right eye. A labial gland biopsy was performed, and the results revealed minor salivary gland tissue with partial acinar atrophy and lymphocytic infiltration in the stroma (≥1 foci/4 mm²) (**Figure 2**). On the basis of patient’s clinical symptoms of ocular and mild dry mouth, positive findings for serum anti-SSA antibodies, the Schirmer’s test results, and focal lymphocytic infiltration of the labial glands, SS was diagnosed in accordance with the 2016 ACR-EULAR classification criteria (1). Given the results of the patient’s cranial MRI and symptoms of chest tightness, the patient underwent coronary angiography and cerebral angiography on the same day. The results revealed 70% stenosis in the proximal segment of the left anterior descending artery (LAD); severe stenosis in the P1 segment of the left posterior cerebral artery, mild stenosis in the M1 segment of the left middle cerebral artery, moderate stenosis in the M1 segment of the right middle cerebral artery, and moderate stenosis in the A2 segment of the right anterior cerebral artery (**Figures 1C-H**). Although vessel wall MRI was recommended to further characterize the intracranial arteriopathy, it was ultimately not performed due to the patient’s personal decision based on financial considerations. The diagnosis relied on the findings from conventional MRA and CTA, as detailed above. Notably, the patient did not have high-risk factors for atherosclerosis such as diabetes, dyslipidemia, obesity, or smoking. Hypertension was detected just two years ago, and blood pressure levels have been well controlled following medication intervention. Nor did the patient’s parents or other first-degree relatives have a history of familial genetic disorders or similar conditions. The diagnostic process after the patient’s admission is shown in **Figure 3**.

**Table 1:** Laboratory investigations.

Test	Result	Reference Range
ANA	1:80	< 1:80
Anti-dsDNA	–	< 20 IU/mL
ANCA (MPO/PR3)	–	–
ESR	9 mm/h	< 20 mm/h
CRP	8.7 mg/L	< 10 mg/L
Serum C3	0.87 g/L	0.80-1.85 g/L
Serum C4	0.32 g/L	0.17-0.48 g/L
ACA IgG	< 5 GPLU/mL	< 8.00 GPLU/mL

ACA, anticardiolipin antibody; ANA, antinuclear antibody; ANCA, antineutrophil cytoplasmic antibodies; CRP, C-reactive protein; dsDNA, double-stranded DNA; ESR, erythrocyte sedimentation rate; GPLU, IgG phospholipid units; MPO, myeloperoxidase; PR3, proteinase 3.

The treatment plan was formulated after extensive discussion with the patient. While the potential role of corticosteroids was explained, the patient, after considering the potential side effects, declined long-term glucocorticoid therapy. This informed decision was formally documented. Consequently, the immunomodulatory regimen was initiated with hydroxychloroquine and total glucosides of paeony (TGPs), alongside secondary preventive measures with aspirin and atorvastatin. The specific protocol included: Hydroxychloroquine Sulfate Tablets 0.2 g orally twice daily, Total Glucosides of Paeony Capsules 0.6 g orally twice daily, Aspirin Enteric-coated Tablets 0.1 g orally once daily, and Atorvastatin Tablets 20 mg orally once daily. Furthermore, the patient reported no recurrence of discomforting symptoms such as dizziness or chest tightness during both the 30-day and 60-day follow-up visits.

## Discussion

pSS often has an insidious onset with highly variable clinical manifestations. The patient, a middle-aged woman from a rural area, presented for her first formal medical contact during this episode. Although she had a history of mild dry mouth, it was not accompanied by difficulty swallowing, patchy tooth loss, dental caries, or salivary gland enlargement, thus escaping her attention. The patient was transferred to our hospital with complaints of dizziness and exercise-induced chest tightness. Previous carotid CTA and brain MRI/MRA performed at an outside hospital revealed abnormal findings. Based on her mild dry mouth symptoms, further tests were conducted during hospitalization, including a serum antinuclear antibody test, Schirmer’s test, and a labial gland biopsy, which confirmed the diagnosis of pSS.

A comprehensive serological evaluation was conducted to exclude alternative causes of multifocal arteriopathy. The negative results for anti-dsDNA and ANCA effectively argue against systemic lupus erythematosus and ANCA-associated vasculitides, respectively. Furthermore, normal complement (C3, C4) levels and the absence of clinical features suggestive of cryoglobulinemia, such as palpable purpura or membranoproliferative glomerulonephritis, make cryoglobulinemic vasculitis a less likely etiology, although it is acknowledged that cryoglobulin testing itself was not performed due to its limited availability—a recognized limitation in this case. Additionally, the normal anticardiolipin antibody (ACA IgG) level provides no serological support for antiphospholipid syndrome. Crucially, inflammatory markers including ESR and CRP were within normal limits, which is inconsistent with the active, systemic inflammation characteristic of large-vessel vasculitis or other highly inflammatory vasculitides such as polyarteritis nodosa. Collectively, despite the absence of cryoglobulin testing, the confluence of these negative serological findings, the absence of corresponding clinical features, and the confirmation of pSS according to international criteria strongly support the conclusion that the observed multifocal cerebral and coronary stenoses are attributable to vasculopathic mechanisms associated with pSS itself.

Studies indicate that patients with primary pSS exhibit a distinct profile of traditional cardiovascular risk factors. Hypercholesterolemia (30%), hypertension (30%), diabetes (27%), and hypertriglyceridemia (22%) are frequently observed, with a significant proportion of patients having at least three risk factors (5). Notably, the prevalence of diabetes and hypertriglyceridemia is higher in pSS patients compared to controls, while hypertension may be underdiagnosed due to less standardized screening (5, 6). Additionally, suboptimal management of hypertension is common (6), and metabolic syndrome is more prevalent in pSS patients than in non-autoimmune controls (7). This patient presented with hypertension as the only traditional cardiovascular risk factor, which is uncommon in pSS populations. Repeated measurements during hospitalization showed normal cholesterol, triglyceride, and fasting blood glucose levels, and blood pressure was well controlled. This suggests that her vascular pathology may be driven primarily by autoimmune-mediated mechanisms, such as endothelial dysfunction or chronic inflammation (8), rather than conventional metabolic risk factors, highlighting the need for individualized cardiovascular risk stratification in pSS patients. Meanwhile, a recent meta-analysis by Karakasis P et al. indicates that pSS is associated with a significantly increased risk of subclinical atherosclerosis, independent of conventional cardiovascular risk factors. The findings—including increased carotid-femoral intima-media thickness, higher prevalence of plaques, endothelial dysfunction, and elevated arterial stiffness—collectively suggest a distinct disease-specific pathophysiology (9). Meta-regression revealed that disease duration and erythrocyte sedimentation rate (ESR), rather than traditional risk factors, were significantly correlated with atherosclerosis severity, highlighting the central role of chronic inflammation and immune-mediated mechanisms. This is consistent with previous studies demonstrating endothelial injury and functional impairment in pSS patients even in the absence of traditional risk factors (2, 8). Therefore, cardiovascular risk management in pSS should extend beyond conventional approaches to include suppression of systemic inflammation and close monitoring of disease activity.

The study conducted by Yong et al. provides critical insights into the differential risks of cardiovascular and cerebrovascular diseases in patients with primary pSS. The pooled results demonstrate a significantly increased overall risk of combined cardiovascular and cerebrovascular events in pSS patients compared to controls (OR = 1.28; 95% CI: 1.11–1.46) (4). However, subgroup analyses reveal a distinct divergence in risk profiles: while the risk of cardiovascular disease (e.g., coronary artery atherosclerosis, acute myocardial infarction) is significantly elevated (OR = 1.30; 95% CI: 1.09–1.55), the risk of cerebrovascular events (including ischemic or hemorrhagic stroke) does not reach statistical significance (OR = 1.31; 95% CI: 0.96–1.79) (4). This suggests that systemic atherosclerosis in pSS may preferentially affect coronary arteries over cerebral vasculature in a broad sense, though specific cerebrovascular phenotypes (e.g., large artery stenosis) were not individually analyzed.

The hypothesis that pSS drives vascular stenosis and atherosclerosis via endothelial dysfunction and chronic inflammation is substantiated by mechanistic insights from related chronic inflammatory diseases. In pSS, persistent autoimmune activation induces a pro-inflammatory state, characterized by elevated cytokines such as TNF-α and IL-6 (10, 11), which directly impair endothelial function by reducing nitric oxide bioavailability and increasing oxidative stress. This endothelial dysfunction manifests as diminished vasodilation, enhanced leukocyte adhesion, and increased vascular permeability, facilitating low-density lipoprotein infiltration and foam cell formation—key events in atherogenesis (12–14). Specifically, in pSS, oxidative stress depletes endothelial antioxidant defenses, leading to apoptosis and dysfunction, while chronic inflammation promotes the expression of adhesive molecule (e.g., ICAM-1, VCAM-1) and monocyte recruitment, accelerating plaque development and instability (14). The consequent progression of atherosclerosis underscores endothelial dysfunction as a critical intermediary, correlating with heightened cardiovascular risk, as evidenced by its predictive value for adverse events in coronary disease (15). This pathway emphasizes the need for therapeutic strategies targeting inflammation and endothelial preservation in pSS to attenuate atherosclerosis.

The cerebral arteriopathy in pSS can present with dynamic, multifocal stenoses affecting both anterior and posterior circulations. Nagahiro et al. described bilateral internal carotid artery occlusions at the C3 level, accompanied by vertebral artery occlusion and extensive moyamoya-like collateralization, as well as the formation of a smoky vascular network (16). Sakata et al. reported progressive M1 segment middle cerebral artery stenosis culminating in occlusion, with contralateral MCA stenosis that exhibited transient reversibility under treatment, suggesting inflammatory vasculopathy rather than fixed atherosclerosis (17). These features align with the extensive vascular involvement observed in our case, which showed simultaneous stenoses in multiple cerebral arteries (including PCA, MCA, and ACA segments) alongside significant coronary artery stenosis. The recurrent and evolving nature of these lesions, as seen in Sakata’s case highlights the role of active immune-mediated vasculitis in driving vascular injury. A recent nationwide cohort study demonstrates that primary pSS is associated with a significantly increased risk of coronary heart disease (adjusted HR = 1.17), independent of traditional cardiovascular risk factors. The risk is further elevated by corticosteroid and NSAID use, highlighting pSS as an independent risk factor for CHD (18). This pattern supports the notion that pSS can lead to severe and diffuse vasculopathy, necessitating heightened clinical vigilance for multisystem arterial involvement.

Current management of pSS is guided by EULAR recommendations, emphasizing a multidisciplinary approach (19). Symptomatic treatment for sicca symptoms includes muscarinic agonists (e.g., pilocarpine), saliva substitutes, and artificial tears. Systemic manifestations, particularly those with moderate to high activity on the ESSDAI, warrant immunomodulatory therapy. Glucocorticoids are used at the lowest effective dose for the shortest duration, supplemented by steroid-sparing agents such as methotrexate, azathioprine, mycophenolate, or leflunomide. Biologics like rituximab (anti-CD20) are reserved for severe, refractory cases, especially those with vasculitis, lymphoma, or progressive interstitial lung disease (19, 20). Recent trials support the efficacy of leflunomide-hydroxychloroquine combination (21), iscalimab (anti-CD40) (22), and ianalumab (VAY736, anti-BAFF-R) (23). Nintedanib is indicated for progressive pulmonary fibrosis. Further studies have demonstrated that long-term hydroxychloroquine use, particularly with high adherence (medication possession ratio, MPR ≥ 0.70), is associated with a significantly reduced risk of coronary artery disease in pSS patients (adjusted HR = 0.49) (24). TGPs have been widely used in China to treat patients with pSS (25). The mechanism may involve its primary active component, paeoniflorin, which inhibits NLRP3 inflammasome activation by regulating the Nrf2/HO-1 axis in submandibular gland cells (26). However, it is important to note that the evidence supporting its use is primarily derived from preclinical studies and regional clinical experience. Large-scale, international randomized controlled trials are needed to fully establish its efficacy and safety profile in the global pSS population.

In this case, TGPs were used as a part of a combination therapy within this specific context. The decision to initiate a regimen of hydroxychloroquine and TGPs, without corticosteroids, was based on the patient’s specific clinical profile and quantitative assessment of lower systemic inflammatory activity (ESSDAI Score < 5). Despite significant vascular stenosis, the absence of elevated inflammatory markers (ESR, CRP), constitutional symptoms, or other organ-threatening manifestations suggested a chronic, immunologically mediated vasculopathy rather than an acute, destructive vasculitis. A step-up approach was adopted, with the plan to escalate therapy to glucocorticoids and/or conventional Disease-Modifying Antirheumatic Drugs (DMARDs) should there be any evidence of disease progression or an inadequate response to the initial regimen. The patient’s rapid and sustained symptomatic resolution on this combination supported this initial strategy.

Additionally, it is worth discussing that the patient’s rural background and the fact that this episode represented her “first formal medical contact” highlight the critical role of socio-structural determinants in the delayed diagnosis and severity of pSS. Rural residents often face compounded barriers to healthcare access, including geographic isolation, limited availability of specialized services, and financial constraints, which collectively delay timely medical consultation (27–29). Furthermore, lower health literacy and educational attainment in rural populations may lead to underrecognition of early or nonspecific symptoms—such as mild xerostomia or vague dizziness—thereby postponing appropriate diagnostic evaluation (30–32). These factors, coupled with a potential lack of regular health monitoring, can allow subclinical vascular inflammation to progress unchecked, resulting in the severe, multifocal arterial involvement observed in this case. This underscores the importance of considering socio-environmental context in the clinical assessment of pSS, particularly in regions with significant rural-urban health disparities.

## Conclusion

This case underscores that pSS can present with significant vascular involvement even in the absence of typical sicca symptoms or classic salivary gland manifestations. Clinicians should maintain a high index of suspicion for an underlying autoimmune etiology in patients, particularly middle-aged women, who present with unexplained multivessel arterial stenoses (cerebral, coronary, or carotid) without conventional cardiovascular risk factors.

Key clinical takeaways include:

- Vigilance for Atypical Presentations: Unexplained dizziness, chest discomfort, or exertional symptoms should prompt consideration of systemic vasculopathy in pSS, even when sicca symptoms are mild or overlooked.
- Systematic Diagnostic Approach: In patients with suggestive vascular imaging findings, comprehensive serological testing (anti-SSA/SSB, ANA), objective ocular evaluation (Schirmer’s test), and histopathological confirmation via minor salivary gland biopsy are essential, regardless of the prominence of oral or ocular symptoms.
- Early Immunomodulatory Intervention: Initiation of disease-modifying agents (e.g., hydroxychloroquine, total glucosides of paeony) alongside conventional antiplatelet and statin therapy may halt disease progression and improve outcomes, as demonstrated by this case’s favorable response.
- Multidisciplinary Management: Collaboration among rheumatology, cardiology, and neurology is critical for timely diagnosis and integrated treatment of pSS-related vasculopathy.

This case reinforces that pSS is not merely a gland-specific disorder but a systemic condition with potentially severe vascular complications. Increased awareness and proactive screening are warranted to mitigate long-term cardiovascular and cerebrovascular risks in this population.

This study has several limitations. Firstly, the follow-up period was relatively short (60 days), and long-term outcomes beyond this point are unknown. Secondly, objective assessment of treatment efficacy was limited to clinical symptom resolution, as repeat vascular imaging (e.g., MRA or CTA) was not performed during follow-up due to patient-related logistical and financial constraints. Consequently, we cannot definitively comment on the anatomical progression or regression of the arterial stenoses. The stability of the patient’s condition is currently inferred from her sustained clinical remission. Thirdly, we were unable to include a formal statement of the patient’s perspective due to her personal decision, despite our sincere efforts to obtain one. We acknowledge this as a limitation in fully capturing the patient experience, as recommended by the CARE guidelines. Future studies with longer follow-up and systematic serial imaging are warranted to validate our initial findings and understand the long-term course of pSS-related vasculopathy.

## Data Availability

The raw data supporting the conclusions of this article will be made available by the authors, without undue reservation.
